# Dissociation in Borderline Personality Disorder: Recent Experimental, Neurobiological Studies, and Implications for Future Research and Treatment

**DOI:** 10.1007/s11920-021-01246-8

**Published:** 2021-04-28

**Authors:** Annegret Krause-Utz, Rachel Frost, Elianne Chatzaki, Dorina Winter, Christian Schmahl, Bernet M. Elzinga

**Affiliations:** 1 Institute of Clinical Psychology, Leiden University, Leiden, The Netherlands; 2 Leiden Institute for Brain and Cognition (LIBC), Leiden, The Netherlands; 3 Department of Psychology, King’s College London, Institute of Psychiatry Psychology & Neuroscience, London, UK; 4 Pain and Psychotherapy Research Lab, University of Koblenz-Landau, Landau, Germany; 5 Department of Psychosomatic Medicine and Psychotherapy, Central Institute of Mental Health Mannheim, Medical Faculty Mannheim, Heidelberg University, Mannheim, Germany

**Keywords:** Dissociation, Trauma, Neuroimaging, Borderline personality disorder, Post-traumatic stress disorder (PTSD), Depersonalization disorder, Dissociative identity disorder, Brain function and structure, Dissociation, Experimental research, Trauma

## Abstract

**Purpose of Review:**

The aim of this review article is to give an overview over recent experimental neurobiological research on dissociation in borderline personality disorder (BPD), in order to inform clinicians and to stimulate further research. First, we introduce basic definitions and models that conceptualize dissociation from a transdiagnostic perspective. Then, we discuss recent findings in BPD.

**Recent Findings:**

Stress-related dissociation is a key symptom of BPD, closely linked to other core domains of the disorder (emotion dysregulation, identity disturbances, and interpersonal disturbances). The understanding of neurobiological correlates of dissociation across different psychiatric disorders (e.g., dissociative disorders, post-traumatic stress disorder) is steadily increasing. At the same time, studies explicitly focusing on dissociation in BPD are still scarce.

**Summary:**

There is evidence for adverse effects of dissociation on affective-cognitive functioning (e.g., interference inhibition), body perception, and psychotherapeutic treatment response in BPD. On the neural level, increased activity in frontal regions (e.g., inferior frontal gyrus) and temporal areas (e.g., inferior and superior temporal gyrus) during symptom provocation tasks and during resting state was observed, although findings are still diverse and need to be replicated. Conceptual differences and methodological differences in study designs and sample characteristics (e.g., comorbidities, trauma history) hinder a straightforward interpretation and comparison of studies. Given the potentially detrimental impact of dissociation in BPD, more research on the topic is strongly needed to deepen the understanding of this complex clinical condition.

## Introduction

Dissociation is a complex transdiagnostic phenomenon, which is highly prevalent in dissociative disorders, e.g., dissociative identity disorder (DID), post-traumatic stress disorder (PTSD), and borderline personality disorder (BPD) [1, 2••]. Over the last decades, psychophysiological, neuropsychological, and neuroimaging research has enhanced the understanding of neurobiological underpinnings of dissociation, even though many ambiguities remain. Compared to the relatively large body of literature on dissociative disorders (e.g., DID) and the dissociative subtype of PTSD, research explicitly focusing on dissociation in BPD remains scarce [3, 4••, 5••]. Given the high comorbidity of BPD with dissociative disorders and PTSD, disentangling disorder-specific effects of dissociation is complicated. Nonetheless, dissociation may affect psychopathological symptom presentation and treatment of psychiatric disorders in different ways [3, 4••, 5••]. Therefore, a more detailed review of the current literature on dissociation in BPD can help to improve the understanding of this severe, complex disorder by stimulating more research.

In this article, we present an overview of recent experimental studies on dissociation in BPD. Building on our previous review [3], we focus on neuroimaging studies, published in 2017 or later. In our present article, we further expand our scope by including experimental research using psychophysiological approaches or investigating the effect of dissociation on body perception and pain processing. All these studies were identified by our search terms in relevant databases (PubMed, PsychInfo, Science Direct, and Web of Science), using combinations of the following keyword groups: borderline personality disorder, dissociation, (e.g., dissociative symptoms, dissociative disorders, trait dissociation); brain (e.g., brain alterations, brain activity), experimental, magnetic resonance imaging, neurobiological, neuroimaging, neurophysiological, neuropsychological, and psychophysiological. The search was limited to articles published in English, in peer-reviewed journals, described studies in human participants and used validated standardized self-report measures of dissociation.

To provide a framework for the interpretation of these findings, we first introduce basic definitions and current conceptualizations of dissociation.

## Definitions, Etiological Models, and Clinical Presentation of Dissociation

Dissociation is a complex and transdiagnostic phenomenon, which has been defined as a “*disruption of and/or discontinuity in the normal, subjective integration of one or more aspects of psychological functioning, including – but not limited to – memory, identity, consciousness, perception, and motor control*” (Spiegel et al., 2011; p. 826) [6]. Disruptions involve a wide range of psychological and somatoform functions and can influence daily functioning in various ways. Psychological aspects of dissociation comprise states of subjective detachment, such as depersonalization and derealization, memory fragmentations including amnesia, and identity disturbances. Somatoform dissociation interferes with bodily functioning, e.g., motor control, body representation, and pain perception [6]. The severity of dissociative symptoms lies on a broad continuum [7]. Milder symptoms, such as absorption, depersonalization, or derealization also occur in non-clinical populations, e.g., due to sleep deprivation, exhaustion, stress, or substance misuse. Severe pathological forms of dissociation may involve the inability to access normally amenable information (e.g., dissociative amnesia) and to control motor processes (e.g., tonic immobilization), as well as involuntary and unwanted intrusions of sensory, affective, and cognitive information into conscious awareness or behavior (dissociative flashbacks) [8]. Individuals differ in their general tendency to experience dissociation (*trait dissociation*), which should be differentiated from acute transient states (*state dissociation*). Acute dissociation typically last for minutes or hours, but may also last for days. Multiple validated measures have been developed to assess state and trait dissociation, including (among others) the Structured Clinical Interview for DSM-IV Dissociative Disorders (SCID-D) [9] and standardized questionnaires, such as the Dissociative Experience Scale (DES, trait dissociation) [10] and the Dissociative Stress Scale (DSS, state dissociation) [11].

### Etiological Models of Dissociation

Controversies about the etiology of dissociation reach back to the beginning of modern psychiatry and psychology (for a more detailed discussion, see e.g., [12–14]). The development of dissociation seems to involve a complex interplay of multiple factors, including genetic factors and neurobiological, temperamental disposition, and environmental factors. With regard to environmental factors, “trauma models” highlight the importance of traumatic life experiences (e.g., [15–20], whereas socio-cognitive models underline the role of social-cultural factors, fantasy proneness, heightened susceptibility, and sleep disturbances [21, 22].

Trauma models propose that dissociation is a (potentially evolutionary-based) defensive mechanism to cope with intolerable overwhelming experiences [8, 23, 24]. Dissociation may serve as a survival strategy to deal with extremely stressful emotions, thoughts, and sensations, especially in response to pervasive threat with low or no chance to escape. Traumatic events may be perceived as a film-like scenario that happens to another person (depersonalization/derealization). Out-of-body experiences, analgesia, and emotional numbing may be a response to intolerable physical and emotional pain, as they may create an inner distance from extremely disturbing experiences that cannot be integrated into existing views of the world, self, and others [16]. During dissociation, salient characteristics of the event might be encoded and stored as separate elements [8], leading to a compartmentalization (fragmentation) of memories, which may later re-occur as unwanted implicit flashbacks memories, e.g., in the context of PTSD [25]. Multiple lines of research have provided empirical evidence for the relationship between trauma and dissociation. In a recent meta-analysis [26••], higher dissociation was linked to adverse childhood experiences, especially severe sexual and physical abuse by caregivers. Earlier age of onset, longer duration of abuse, and parental abuse significantly predicted higher levels of dissociation [26••].

However, the trauma model has been frequently debated and different views exist on the mechanisms that underlie the development of dissociative disorders, especially DID [12]. The socio-cognitive model questions the direct causal relationship between trauma and DID and highlights the role of sociocultural expectations or sleeping problems. Questioning techniques and media influence are assumed to contribute to the way individuals with DID express their experiences, such as emotional instability, identity problems, and impulsive behavior [22]. Up to now, there is no agreement on the etiology of dissociation [21, 22] and the etiology may differ from person to person. The neurobiological models, described in more detail below, offer hypotheses as to how potential etiological factors, such as trauma, may have altered brain networks involved in dissociation [17]. Before we turn to these models, we describe how dissociation can have diverse clinical presentations, as different symptoms may be associated with different neurobiological alterations.

### Clinical Presentations

Pathological dissociation is a feature of several psychiatric disorders. A recent meta-analysis found that dissociative experiences are most prevalent in dissociative disorders (DID, dissociative amnesia, depersonalization/derealization disorder, dissociative fugue, and dissociative disorder not otherwise specified), followed by PTSD, BPD, and conversion disorder [2••]. Dissociation can also occur as a symptom of schizophrenia [27], major depressive disorder [28], bipolar disorder [29], and obsessive-compulsive disorder [30]. The differentiation between psychotic and dissociative symptoms can be challenging [31, 32]. Many patients with severe dissociative symptoms have a long history of hospitalizations, misdiagnoses, and inefficient pharmacological treatment [33], and it often takes years for patients to find adequate treatment [34]. Understanding the psychopathological context in which dissociation occurs (e.g., degree of emotion dysregulation and suicidality) may improve its treatment [35, 36].

In BPD, stress-related dissociation is one of the core diagnostic features [1]. Up to 80% of patients experience transient dissociative symptoms. Research suggests that dissociative symptoms are most severe in a subgroup of patients with higher overall symptom severity [1, 37] and more severe traumatic experiences [26••, 38].

Dissociation has been linked to other BPD core features, especially to emotion dysregulation and identity disturbances [39]. Emotion dysregulation includes a predisposition towards intense, emotional reactions, and maladaptive emotion regulation strategies, e.g., suicidal behavior, non-suicidal self-injury (NSSI), substance abuse, spending sprees, and risky sexual encounters [40, 41]. The strength, frequency, and intensity of dissociation correlate with emotional distress [42] and impulsive decision-making [43]. Dissociation may also exaggerate difficulties identifying emotions and being aware of them [44]. Reducing emotional distress and terminating dissociation are main motives for NSSI in adults with BPD [45, 46], possibly related to reduced pain processing and analgesia during dissociative states [47]. Dissociation has also been associated with more intense suicidal ideation in adolescents with BPD features, although it did not necessarily contribute to self-injury in this adolescent population [48].

Disturbances in identity are another core domain of BPD [49–51]. Individuals with the disorder experience rapid changes in identity, which is often experienced as incoherent, inconsistent, vague, or fragmented, accompanied by objective incoherencies in thought, feeling, and behavior [49]. These identity disturbances show considerable overlap with features of identity disorders and dissociative disorders [52]. It has been proposed that identity disturbance in BPD is less stable and more subtle than in DID [53]. However, a lack of subjective coherence along with an objective incoherence in thoughts, feelings, and behavior was also found to distinguish BPD from other mental disorders [49]. In BPD, identity/sense of self is closely related to low self-esteem, which is highly unstable under daily life conditions [50, 54]. Along with a negative self-esteem, individuals with BPD often report a negative body perception [51, 55], which has been linked to child sexual abuse [56].

Interpersonal disturbances (e.g., rejection sensitivity, marked mistrust, and a strong ambitendency between an intense need for closeness and a need for autonomy) are the third core domain of BPD [57–60]. Individuals with BPD are not only more sensitive to negative social clues, such as angry faces [61] but also have problems in detecting and memorizing positive social signals and events [57, 62, 63]. A negativity bias, e.g., when evaluating facial expressions may contribute to these difficulties [61, 64, 65] and may make individuals with BPD more susceptible to emotional interference [64]. Distractibility by social cues (e.g., faces, interpersonal scenes) has been linked to acute dissociation in BPD [66]. However, it remains unclear whether dissociation influences the perception of social stimuli (e.g., contributes to a negativity bias) in BPD. A recent study in individuals who experienced childhood maltreatment did not reveal evidence for a significant correlation between dissociation and deficits in the interpretation of neutral facial expressions [67]; respective studies in BPD are still needed. In general, dissociation may also contribute to increased risk of re-victimization in individuals who experienced child sexual abuse. Along with maladaptive cognitive coping styles (e.g., self-blame) dissociation was found to mediate this link [68, 69], which needs to be replicated in prospective studies.

Importantly, acute dissociation predicted poor treatment outcome in BPD patients (with and without PTSD) [70, 71], which may not be the case for trait dissociation and PTSD patients without BPD (see meta-analysis by Hoeboer et al., 2020 [72••]). In two different treatment studies, patients with BPD and higher levels of dissociation had relatively poor treatment response to dialectical behavior therapy (DBT) [70, 71]. A possible reason for this link may be that acute dissociation interferes with emotional learning (e.g., during exposure treatment) in BPD [66, 73–75]. Patients with the disorder who reported acute dissociation, showed impaired acquisition during a differential aversive delay-conditioning paradigm [74] and during an operant conditioning task [76]. In the latter study, patients were exposed to aversive and neutral stimuli while performing a task combining learning acquisition and reversal. Higher dissociation, along with increased emotional arousal, was related to worse acquisition, but not reversal learning in BPD. Dissociation may particularly interfere with the acquisition of new information in a stressful context. A recent systematic review suggests that impaired attention, executive functioning, memory, and social cognition can be found across different psychiatric disorders with high trait dissociation [77]. However, acute dissociation may affect executive functions in these disorders in different ways. In BPD, dissociation was associated with impaired working memory [66] and other executive functions implicated in goal-directed behavior [66, 73, 75, 78], whereas it might be linked to improved working memory in DID (e.g., [79]). This raises the question whether the observed effects may be specific to certain disorders or subsamples. More research is needed to disentangle the effects of dissociation on treatment in the context of BPD, PTSD, and DID.

Adding to this debate, it remains unclear if certain alterations in affective-cognitive processing during dissociation are due to a history of complex trauma (e.g., severe abuse and neglect), which is frequent in BPD and other disorders associated with dissociation. In 2018, the 11th edition of the World Health Organization’s International Classification of Diseases (ICD-11) [80] introduced the diagnosis of complex PTSD (CPTSD). As a “sibling disorder” of PTSD, CPTSD involves “classical” PTSD symptoms (intrusive re-experiencing, avoidance of traumatic reminders, hyperarousal) and two symptoms from each of three domains of Disturbances of Self-Organization (DSO): [1] emotional numbing and dysregulation [2••], self-perceptions as guilty or worthless [3], and emotional detachment in relationships. There is potential symptom overlap between CPTSD, DSM-based PTSD, BPD, and DID [81]. While there is still a lack of consensus regarding the validity of the CPTSD diagnosis, research suggests that it detects a group of individuals with significant impairment who would not receive a diagnosis under the DSM-5 [82]. Compared to PTSD, individuals with CPTSD more often report multiple forms of trauma that are predominantly interpersonal in nature [83]. With respect to dissociation, those with CPTSD report significantly higher levels of dissociative experiences compared to those with PTSD (Cohen’s *d* = 1.04) and compared to those with no diagnosis (*d* = 1.44) in a highly traumatized clinical sample [84].

Both CPTSD and BPD encompass difficulties in emotion regulation, self-concept, and interpersonal relationships. While BPD diagnosis does not require an index trauma, symptoms of both disorders may likely co-occur [85]. Therefore, a growing number of studies have aimed to investigate how CPTSD and BPD can be empirically distinguished among trauma-exposed populations [81]. In this context, further research needs to determine if dissociation is a risk factor for the development and outcome of CPTSD [84] and how this may be differentiated from dissociation in other disorders, such as PTSD [1], as well as BPD [81].

## Neurobiological Models and Transdiagnostic Research on Dissociation

Several models have proposed that pathological dissociation coincides with a distinct pattern of neurobiological alterations, such as an increased recruitment of brain regions implicated in the control of emotions and somatosensory input, as well as dampened autonomic arousal.

In 1998, Sierra and Berrios proposed a corticolimbic-disconnection model [86], which suggests that a “disconnection” of corticolimbic brain regions contributes to symptoms of depersonalization, such as numbing, analgesia, hypervigilance, and emptiness of thoughts.

It is assumed that depersonalization involves an increased activation of medial and dorsolateral prefrontal cortices (areas implicated in cognitive control and arousal modulation). Both directly and indirectly, via the anterior cingulate cortex (ACC), these regions are assumed to dampen activity in the amygdala. In other words, an increased interplay of these regions is assumed to lead to a dampening of autonomic arousal. Decreased activity of the amygdala, which is crucial to the initiation of stress and fear responses, may be associated with a shutting down of the affective system [87]. Evidence for this model stems from research in depersonalization disorder [87–89]. In addition to the abovementioned regions, the dorsomedial prefrontal cortex, posterior insula [90], and posterior cingulate [91] have been implicated in altered inward-directed processing (e.g., self-referential processing), which may contribute to the proposed shutdown of the affective system during depersonalization.

An autonomic “shutdown” during dissociation, associated with increased parasympathetic activity, has also been proposed by another model, the defense cascade model by Schauer and Elbert (2010) [23]. We refer to this model in more detail below, in the context of psychophysiological research on dissociation in BPD.

Based on the idea that dissociative symptoms, such as distortions in time, thought, body, and emotions, are distinct “trauma-related states of consciousness” [31], it has been proposed that they are associated with a distinct psychophysiological and neural profile [17, 18]. With respect to PTSD and its dissociative subtype (D-PTSD) [1], Lanius and colleagues [17] proposed that D-PTSD can be differentiated from PTSD by a distinct pattern of neural activity [31], involving increased frontal activation (in dorsal/rostral ACC, mPFC) and dampened limbic activity (in the amygdala and insula). In addition, alterations in the thalamus (filtering sensory input from both subcortical limbic regions and frontal areas) [18] and regions implicated in defensive coping responses, such as the periaqueductal gray (PAG) [92] and the superior colliculus [93] have been implicated in the dissociative subtype. Reduced sensory processing may underlie reduced attention and arousal, which may lead to progressive cognitive dysfunction [77]. A study using stochastic dynamic causal modeling of resting-state functional connectivity (RS-FC) in D-PTSD [94] found greater amygdala FC with prefrontal regions involved in emotion regulation (middle frontal gyrus, medial frontal gyrus). These findings are in line with the idea that dissociation may be a self-regulatory response to cope with overwhelming emotions. In another study by this group [95], patients with D-PTSD showed a predominant pattern of top-down emotion regulation from the ventromedial prefrontal cortex to the amygdala and PAG and from the amygdala to the PAG. Additionally, a stronger coupling of the (bilateral anterior, left mid, and left posterior) insula to the left basolateral amygdala complex was found in D-PTSD [96], which correlated with depersonalization/derealization symptoms and PTSD symptom severity. A functional magnetic resonance imaging study by Felmingham and colleagues [97] found enhanced ventral prefrontal cortex activation (suggesting enhanced prefrontal control) for threatening information that was presented on a supraliminal (conscious) level. When threatening stimuli were presented on a non-conscious level, PTSD patients with high dissociation showed increased activity in the bilateral amygdala, insula and left thalamus compared to patients without dissociation. These findings are mostly in line with previous findings in D-PTSD [17] and suggest that enhanced prefrontal control may be a conscious coping strategy, which may not function during non-conscious threat processing. A more recent study by this group [98] revealed a positive correlation between activity in the right insula during supraliminal (conscious) threat processing and dissociative symptoms in patients with CPTSD.

Resembling findings in PTSD, two studies in DID observed lower activity in cingulate gyrus, parietal cortex, and para-hippocampus when patients reported voluntary access to traumatic memories, whereas the opposite response pattern was found during dissociative amnesia [99, 100]. These findings suggest that the abovementioned processes are likely transdiagnostic in nature.

Indeed, a recent systematic review by Lotfinia, Soorgi, Mertens, and Daniels (2020) [4••] that included 33 functional and structural neuroimaging studies across different disorders found evidence for transdiagnostic brain alterations in frontal and temporal regions, with the closest overlap for PTSD and DID. However, studies included in the systematic review [4••] were quite diverse, covering widespread clusters of neural alterations, and findings should be considered preliminary, as replication studies are strongly needed.

Another (more extensive) systematic review by Roydeva and Reinders (2020) [5••], which included 205 unique studies, suggests that functional alterations in the dorsomedial and dorsolateral prefrontal cortex, bilateral superior frontal regions, (anterior) cingulate, posterior association areas, and basal ganglia may be seen as neurofunctional biomarkers of pathological dissociation across different psychiatric disorders. One of the most consistent neurofunctional findings was enhanced activity of the inferior frontal gyrus and medial prefrontal cortex during symptom provocation tasks. With respect to brain structure, a relatively consistent pattern of decreased volumes in the hippocampus, basal ganglia, and thalamus emerged.

Nonetheless, it is not entirely clear whether the aforementioned models and empirical findings are representative of the variety of dissociative symptoms in different mental disorders [4••, 5••]. As previously mentioned, dissociation in BPD seems to be particularly related to acute states of high arousal and disturbed emotional memory and learning [42, 66, 73–75], whereas in DID the opposite pattern was observed [79]. Therefore, functional alterations in BPD may be more closely related to temporary changes and more clearly detected during acute dissociation, as further discussed below.

## Neuroimaging Studies on Dissociation in BPD

In general, neuroimaging research in BPD is rapidly growing. The constant development of neuroimaging methods and analytical techniques has increased knowledge about possible neurobiological underpinnings. Disturbances in corticolimbic circuitry involving the amygdala, hippocampus, insula, anterior cingulate, orbitofrontal cortex, and medial prefrontal cortex have been associated with problems in emotion regulation, interpersonal disturbances, and disturbed identity [101, 102].

Amygdala hyper-reactivity to threat-related stimuli has been trans-diagnostically linked to increased emotional responsiveness. A recent meta-analysis concluded that amygdala hyper-reactivity is more pronounced in BPD patients than in both healthy and depressed samples [103]. Reduced amygdala habituation to repeated negative stimuli in BPD is a consistent finding ([104], see also [102]), and seems to be related to adverse childhood experiences [105]. Importantly, increased amygdala response to negative versus neutral images predicted poor individual treatment response in BPD [106]. In this recent proof-of-principle study, multimodal MRI (functional MRI during three different emotion regulation tasks and structural MRI), demographic, and clinical data was used to predict individual therapy response for DBT, using random forest classification analysis. Increased amygdala and para-hippocampal activation during a cognitive reappraisal task (emotional challenge and regulation), along with BPD severity and amygdala gray matter volume, predicted individual treatment response. Moreover, there is evidence that the amygdala may be a promising target for neurofeedback training in BPD [107, 108]. After real-time fMRI neurofeedback training of amygdala hemodynamic activity, participants improved downregulation of their amygdala blood oxygen-level-dependent (BOLD) response, showed a decrease in emotion-modulated startle to negative pictures, and reported less affective instability in their daily life [107].

While it has been proposed that diminished prefrontal control of the amygdala underlies clinically observed problems in emotion regulation and impulse control, this model has been challenged due to methodological problems, e.g., a lack of spatial and disease specificity [109, 110]. Reduced activity in dorsolateral and orbitofrontal regions has been observed in subgroups of patients who show anger-related aggression [111], but its role in deficient emotion dysregulation in BPD remains elusive [109].

To our knowledge, only few neuroimaging studies in BPD focused explicitly on brain alterations linked to dissociation. Table 1 provides an overview of studies, identified by our literature search and published between January 2017 and December 2020. This table illustrates that studies are not only rare bur also very diverse, in terms of sample characteristics (e.g., comorbidities, trauma histories, medication status), methods (e.g., functional or structural magnetic resonance imaging, electroencephalography), designs (e.g., symptom provocation tasks, resting state), and analyses (e.g., seed-based region of interest analysis, functional connectivity analysis), which hinders a straightforward comparison and interpretation of findings.

**Table 1:** Overview of experimental studies on dissociation in borderline personality disorder, identified by our literature search as published between 01/2017 and 12/2020

Authors, year of publication	Sample (groups, sample size), gender	Psychotropic medication status	Comorbidities and trauma history in the patient sample	Method and experimental design	Measures of dissociation (trait/state, time)	Key findings concerning dissociation
Neuroimaging studies on dissociation						
Baczkowski et al., (2017)	• Groups: - BPD (*n* = 48) - Non-patients (NPC, *n* = 48) - Cluster-C PD (CPD, *n* = 31) • Gender: Female	Most patients received antidepressants, and further received antipsychotics, hypnotics, or mood stabilizers.	All patients reported a history of childhood trauma (including emotional, physical and sexual abuse, emotional/physical neglect). Comorbidities predominantly with major depressive disorder (MDD) and substance abuse.	Resting state (RS) fMRI was acquired to investigate effects of effortful emotion regulation on amygdala functional connectivity in BPD.	Self-reported dissociation (DES) assessed prior to and immediately after scanning.	Unlike controls, BPD patients did not show an increase of amygdala resting-state functional connectivity with medial, dorsolateral, ventrolateral PFC and superior temporal gyrus after effortful emotion regulation. Dissociation did not correlate with change of amygdala RSFC in BPD.
Krause-Utz et al., (2018)	• Groups: - BPD patients with acute dissociation (dissociation script, BPDd, *n* = 17) - Patients exposed to neutral script (BPDn, *n* = 12) - HC (*n* = 18) • Gender: Female	Free of psychotropic medication for at least 4 weeks prior to study.	All patients reported at least one type of severe to extreme childhood trauma. 5 patients in the BPDn group and 7 in the BPDd group met criteria for current PTSD. Comorbidity with current (other) anxiety and eating disorders. Lifetime psychotic disorder, bipolar-I disorder, mental retardation, and substance abuse 6 months prior to scan were excluded.	fMRI to measure changes in BOLD signal, combining script-driven imagery (experimentally induces dissociation) with a subsequent emotional working memory task (EWMT, with socio-emotional distractors).	Dissociation was induced in 17 patients, while 12 patients and 18 HC were exposed to a personalized neutral script. Self-reported trait dissociation (DES) and state dissociation at baseline, after script and after EWMT.	BPDd showed WM deficits, reduced bilateral amygdala activity (across conditions) and reduced left cuneus, lingual gyrus, and posterior cingulate activity (during negative distractors) compared to BPDn. Inferior frontal gyrus activity was higher in both BPD groups than in HC. BPDd showed a stronger coupling of amygdala with right superior/ middle temporal gyrus, right middle occipital gyrus, left inferior parietal lobule, and left claustrum than BPDn and HC.
Popkirov et al., (2019)	• Groups: - BPD (*n* = 26) - HC (*n* = 26) • Gender: Female	Some patients received antidepressants (*n* = 11), antipsychotics (*n* = 6), anticonvulsants (*n* = 3) and other psychoactive drugs (*n* = 2).	All patients reported a history of childhood trauma (including emotional, physical and sexual abuse, neglect). Some patients met criteria for depression (*n* = 17), phobic/anxiety disorder (*n* = 3), PTSD (*n* = 5), or substance abuse (*n* = 6).	EEG recordings before (pre) and after (post) mood induction using aversive IAPS pictures (50 negative/50 neutral) to measure frontal electroencephalographic asymmetry (FEA) as a trait and/or state parameter of emotion regulation.	Self-reported dissociation (DES).	Changes in FEA in BPD and healthy controls while negative pictures were presented (slight but significant shift from left- to right-sided asymmetry over prefrontal electrodes). Baseline FEA correlated significantly with childhood trauma severity (CTQ) and trait dissociation (DES) in patients with BPD.
Zaehringer et al., (2019)	• Group: BPD (*n* = 24) • Gender: Female	All participants were on stable medication, including SSRI (*n* = 3), SNRI (*n* = 4), Tricyclic (*n* = 3) other antidepressants (*n* = 3), neuroleptics (*n* = 5) and anticonvulsants (*n* = 2); 10 patients were un-medicated.	Some patients met criteria for current MDD (*n* = 6), lifetime MDD (*n* = 22), PTSD (*n* = 6). Other comorbidities were dysthymia, double depression, panic disorder, social phobia disorder, specific phobia, and eating disorder. Alcohol or substance abuse 6 months prior to scan, lifetime psychotic disorder, bipolar affective disorder and mental retardation were excluded.	Real-time fMRI-based neurofeedback training consisting of four sessions in which participants viewed aversive pictures and received feedback from a thermometer displaying amygdala BOLD signals during an Emotional working memory task and a Backward Masking Task.	Self-reported dissociation (DSS).	After training, participants reported a decrease of BPD symptoms and showed a decrease in emotion-modulated startle to negative pictures. A reduction of dissociation was observed which did not reach significance.
Psychophysiological studies on dissociation						
Bischescu-Burian et al., (2017)	BPD (*n* = 13), BPD with high peritraumatic dissociation (PD, *n* = 15) and healthy controls (HC, *n* = 15), all female.	Free of psychotropic medication for at least 1 week prior to physiological assessment.	Patients reported childhood trauma. Some patients met criteria for PTSD or dissociative disorder. Other comorbidities were affective disorders, anxiety disorder, and eating disorders. Exclusion of schizophrenia and substance abuse.	Participants were exposed to personalized script-driven imagery (consisting of 3 script presentations) with traumatic and everyday neutral events. Emotional and psychophysiological reactions (heart rate (HR) and skin conductance (SCR)) were assessed during the experiment.	Self-reported trait dissociation (DES) and peritraumatic dissociation experiences (PDEQ).	BPD patients with high PD showed a significant HR decline during imagery of traumatic events (versus increased HR in the other groups); HR responses were predicted by PD, but not by measures of state or trait dissociation. Groups did not differ in SCR.
Koenig et al., (2018)	Adolescents (13–19 years) with BPD (*n* = 30), healthy controls (*n* = 34) and psychiatric clinical controls (CC, *n* = 53) participated in the trial, all female.	Some of the BPD and CC patients received medication including anti-depressives (*n* = 6), stimulants (*n* = 2), neuroleptics, (*n* = 3) and other (*n* = 4).	BPD and CC patients reported sexual (*n* = 13) and physical abuse (*n* = 15). The BPD and CC patients presented with a range of axis I and axis II disorders. Exclusion criteria included current drug or alcohol dependence, lifetime schizophrenia, schizoaffective disorder, bipolar disorder, pervasive developmental disorder or any neurological disease.	Participants listened to startle-probes (78-dB, 1000 ms, 1000 Hz) while heart rate (HR) and skin conductance (SCR) were continuously recorded.	Self-report dissociation scale for adolescents (DES-A).	HR significantly differed between the CC and BPD groups. HR was also associated with number of BPD diagnostic criteria and with symptoms of dissociation. Delayed HR habituation across probes was associated with greater BPD symptom severity.
Krause-Utz et al., (2018)	BPD (BPD, *n* = 37), BPD with comorbid PTSD (BPD + PTSD, *n* = 20) and healthy controls (*n* = 27), all female.	*n* = 23 patients (BPD: *n* = 15; BPD + PTSD: *n* = 8) received psychotropic medication, including SSRI, tricyclic antidepressants, and antipsychotic medication.	All patients reported childhood trauma. Some patients had comorbid MDD (*n* = 21), or anxiety disorders (*n* = 13). Exclusion criteria for patients were lifetime history of bipolar disorder, psychotic disorder, acute life-threatening suicidal crisis, mental deficiency, and severe organic disorder.	Participants performed a cognitive reappraisal (emotion regulation) task with neutral, positive, and negative images. Participants were instructed to either attend these pictures or to down-regulate their upcoming emotions. Acute arousal, wellbeing, dissociation, and electrocardiogram data were assessed.	Self-reported dissociation (Dissociative Tension Scale, DSS4).	Independent of instruction and picture valence, both patient groups reported higher arousal, lower wellbeing, and more dissociation than HC. BPD + PTSD showed significantly lower HF-HRV than the other groups. In BPD + PTSD, higher state dissociation at baseline predicted higher HF-HRV during down-regulating vs. attending negative pictures.
Body ownership						
Löffler et al., (2020)	Patients with acute BPD (BPD-C, *n* = 26), remitted BPD (BPD-R, *n* = 22) and healthy controls (HC, *n* = 20), all female.	Apart from selective serotonin reuptake inhibitors, patients were free of psychotropic medication.	Exclusion of lifetime bipolar-I disorder or schizophrenia, mental retardation, and substance dependency disorder within the last year.	Body ownership for 25 body areas, relations to dissociation and other relevant BPD markers were assessed. Participants were asked to close their eyes, sit relaxed, and rate the state degree of belongingness for a given body area. All 25 body areas were read out loud. Participants rated each item verbally.	Self-reported trait (German DES)	Patients with acute BPD showed reduced body ownership experiences compared to HC, while they did not differ from those with BPD in remission. In acute BPD, reduced body ownership was significantly related to dissociation when controlling for other BPD core features.
Pain processing						
Chung et al., (2020)	Patients with acute BPD (BPD-C, *n* = 25), remitted BPD (BPD-R, *n* = 20) and healthy controls (*n* = 24), all female.	*n* = 3 BPD-C patients and *n* = 1 BPD-R patient received anti-depressant medication (SSRIs).	*n* = 9 patients in the BPD-C group met criteria for current PTSD, and 10 patients from the BPD-C and 5 from the BPD-R group met criteria for lifetime PTSD. Comorbidity with current and lifetime depression, current anxiety disorders, and phobias was evident. Lifetime diagnosis of schizophrenia, bipolar-I disorder, substance dependence 2 years prior to study or current substance abuse were excluded.	Script-driven imagery to induce dissociation: participants were exposed to a personalized dissociation-inducing script (vs. to a neutral script). Warm perception thresholds (WPT) and heat pain thresholds (HPT) were assessed.	Self-reported dissociation on the DSS4, assessed prior to and after the script.	Compared to HC, both BPD-C and BPD-R showed enhanced dissociation along with pain hyposensitivity (higher HPT levels) in the stress condition. In BPD-C (but not in BPD-R), a significant association between dissociation proneness and hyposensitivity to pain (suggesting analgesia) was observed.
Defrin et al., (2019)	BPD (*n* = 22) and HC (*n* = 33), all female.	*n* = 18 BPD patients received psychotropic medication (antidepressants: *n* = 15; anxiolytics: *n* = 11).	Comorbid anxiety disorders (*n* = 8), MDD (*n* = 7), attention deficit hyperactivity disorder (*n* = 3), eating disorder (*n* = 3) or PTSD (*n* = 2). Exclusion of neurological and systemic diseases, or lifetime psychosis substance abuse.	Pain modulation measurements of warmth sensation threshold (WST) and heat pain threshold (HPT), conditioned pain modulation (CPM) and temporal summation of heat pain (TSP).	Self-reported Dissociation (DES).	Patients with BPD showed higher WST and HPT, suggesting generalized hyposensitivity and more efficient pain inhibition capabilities, compared with HC. No significant correlation between HPT and dissociation in BPD.

*BOLD*, blood oxygen level-dependent; *BPD*, borderline personality disorder; *BPD-C*, acute BPD; *BPD-R*, remitted BPD; *CPM*, conditioned pain modulation; *CTQ*, Childhood Trauma Questionnaire; *DES*, dissociation experience scale; *DES-A*, dissociation experience scale adolescent; *DSS21*, Dissociation Stress Scale 21 items version; *DSS4*, Dissociation Tension Scale 4 items version; *EWMT*, emotional working memory task; *FEA*, frontal electroencephalographic asymmetry; *fMRI*, functional magnetic resonance imaging; *HC*, healthy controls; *HPT*, heat pain threshold; *HRV*, heart rate variability; *IAPS*, international affective picture system; *MDD*, major depressive disorder; *PDEQ*, Peritraumatic Dissociation Experiences Questionnaire; *PD*, personality disorder; *PFC*, prefrontal cortex; *PTSD*, post-traumatic stress disorder; *RSFC*, resting-state functional connectivity; *SSRI*, selective serotonin reuptake inhibitors; *TSP*, temporal summation of heat pain

Previous studies used functional magnetic resonance imaging (fMRI) during symptom provocation tasks or during resting state to investigate brain activity in BPD patients with high dissociation. Findings of these studies most consistently pointed to increased activity in frontal areas (inferior and middle frontal gyrus, superior frontal regions) and reduced activation in temporal areas (inferior and superior temporal gyrus) during symptom provocation and rest. Some studies also found lower activity in the amygdala during presentation of aversive pictures in patients who reported higher dissociation. However, findings are diverse and some studies did not find significant links between altered task-related or resting-state brain activity and dissociation (for a more detailed description see [3] and Table 1).

To our knowledge, so far, only three neuroimaging studies in BPD investigated the effect of experimentally induced acute dissociation on neural processing, using a script-driven imagery paradigm [66, 75, 112]. In this paradigm, patients create a narrative of a personal situation in which dissociative experiences occurred. During the experiment, patients listen to this script to recall the autobiographical situation, while behavioral, neuropsychological, psychophysiological, and/or neural reactivity are measured and compared to patients who listen to an emotionally neutral script. Across three different studies, this experimental paradigm led to a significant increase in self-reported dissociation [66, 75, 112]. Patients who listened to a dissociation script also reported lower pain sensitivity [112], confirming earlier findings [47]. Two of these studies combined script-driven imagery with neuropsychological tasks measuring emotional interference inhibition, i.e., the Emotional Stroop Task (EST) and the Emotional Working Memory Task (EWMT). During the EST, patients who had been exposed to a dissociation script showed impaired task performance (overall slower and less accurate responses), impaired interference inhibition for negative versus neutral words, and altered activity in regions implicated in interference inhibition (fusiform gyrus, left inferior frontal gyrus, parietal cortices) [75]. More recently, Krause-Utz et al. (2018) [66] combined script-driven imagery with an EWMT. Patients who listened to a dissociation script later showed impairments in working memory after distraction by social information (pictures of interpersonal violence versus neutral interpersonal scenes) and in distractor-free trials [66]. On the neural level, patients with acute dissociation showed increased activity in the inferior frontal gyrus, which is consistent with the other two script-driven imagery studies [75, 112]. In addition, patients who performed the EWMT after listening to the dissociation script showed reduced amygdala activity and reduced left cuneus, lingual gyrus, and posterior cingulate activity [66].

The complexity of dissociative symptoms and the broad range of functions that they affect render it unlikely that effects can be traced down to a few localized brain regions. Several studies used functional connectivity analysis to investigate dynamic interactions between brain regions within large-scale brain networks. During rest, trait dissociation (DES) predicted stronger resting-state functional connectivity (RSFC) of the amygdala with the dorsolateral prefrontal cortex, and reduced RSFC with occipital fusiform areas in BPD [113]. In another study [114], acute dissociation was linked to a stronger coupling of the amygdala with the insula, ACC, and thalamus during the presentation of aversive distractors. In another study, dissociation increased after scanning, but these changes did not predict changes in amygdala RSFC after an emotion regulation task [115]. Differences in sample characteristics (e.g., medicated [115] versus un-medicated [113]) may partly contribute to these discrepancies.

A recent study by Popkirov and colleagues (2019) [116••] used electroencephalography (EEG) to investigate frontal electroencephalographic asymmetry (an indicator of emotion regulation) and its link to somatoform dissociation (conversion symptoms, as assessed by the German version of the DES). Patients with BPD and healthy controls were exposed to highly aversive pictures mixed with neutral pictures. Both groups showed a significant shift from left- to right-sided asymmetry during the experiment, which suggests effortful emotion regulation. Interestingly, frontal electroencephalographic asymmetry at baseline was significantly correlated to childhood trauma severity and dissociative tendencies (DES) in the BPD group. In the context of earlier neuroimaging findings, these results provide preliminary evidence for a link between dissociation (conversion symptoms) and emotion regulation. However, more studies are needed to replicate these findings before firm conclusions can be drawn.

In a similar vein, only a few BPD studies investigated associations between dissociation and altered brain structure. Two studies found preliminary evidence for larger volumes of the right precuneus and left postcentral gyrus [117] and larger gray matter volumes in the middle and superior temporal gyrus [118]. More research is needed to understand if dissociation is associated with altered brain structure in BPD.

So far, the most consistent findings seem to be an increase in frontal activity (e.g., in inferior frontal gyrus, superior frontal regions) and reduced activation in temporal areas (inferior and superior temporal gyrus). Altered activity in the inferior and middle frontal gyrus and superior frontal regions was also observed in D-PTSD [97], DID [99, 119], conversion disorder [120], and depersonalization [86]. The abovementioned recent systematic review by Lotfinia and colleagues [4••] suggests that alterations in these frontal and temporal may be a transdiagnostic neurobiological marker of dissociation. The systematic review by Roydeva and Reinders [5••] also found evidence for functional alterations in the dorsomedial and dorsolateral prefrontal cortex, bilateral superior frontal regions, (anterior) cingulate, posterior association areas, and basal ganglia across different psychiatric groups. Whether certain alterations (e.g., reduced amygdala activity) may be specific to acute stress-related dissociation in BPD is an open research question that needs to be investigated in future studies, comparing patients with BPD to other clinical groups with dissociation. A more specific assessment of dissociation and a careful assessment of potentially confounding variables (e.g., trauma history) may be a helpful step in this direction.

## Other Recent Research on Dissociation in BPD

Since research on dissociation in BPD is scarce, we broadened the scope of our current review, including studies that used other experimental approaches (psychophysiological measures, pain processing, body perception) to study dissociation. While these studies deviate from the main focus of our previous review (neuroimaging studies) [3], they might offer important input for future neuroimaging research on dissociation in BPD. Studies that met the abovementioned search terms in relevant databases (PubMed, PsychInfo, Science Direct, Web of Science) are summarized in Table 1.

### Psychophysiological Studies

Several neurobiological models propose that altered neural activity during dissociation is accompanied by psychophysiological changes in heart rate (variability), skin conductance response (SCR)/electrodermal activity, blood pressure, and fear-potentiated startle response. The aforementioned corticolimbic-disconnection model [86] proposes that increased prefrontal modulation of amygdala activity is associated with dampened autonomic arousal, which may show in reduced startle response [73, 121]. The defense cascade model by Schauer and Elbert (2010) [23] proposes a sequence of fear responses that escalate as a function of proximity of threat. More specifically, after an initial increase in sympathetic activation, the organism is assumed to respond with a parasympathetically dominated “shutdown” once the threat becomes too close and unpreventable (i.e., possibilities of defense or escape are not available or fruitless). Increased parasympathetic activity during dissociation has also been linked to a drop in heart rate [17, 121]. Across psychiatric groups with dissociative features, findings of psychophysiological studies were mixed and too diverse to identify potential neurofunctional biomarkers of pathological dissociation [5••].

In BPD specifically, previous studies found reduced startle response while listening to startling tones [73] and stressful scripts [122]. It has been proposed that low autonomic arousal may reflect an interfering effect of dissociation on emotional processing in BPD [123]. Patients with BPD who reported high acute dissociation also showed diminished SCR during a fear conditioning paradigm [74] and stressful scripts [122]. More recently, Bichescu-Burian and colleagues (2017) [124••] used the abovementioned script-driven imagery paradigm to investigate psychophysiological responses to recall of traumatic events. Heart rate and skin conductance responses (SCR) in BPD patients who had experienced high levels of peritraumatic dissociation were compared to BPD patients with low PD and healthy controls. Patients with high PD showed a significant heart rate decline during the imagery of personal traumatic events compared to the two other groups, while no differences in SCR were found. Increases in trauma-evoked heart rate were predicted by peritraumatic dissociation but not by measures of state or trait dissociation. However, sample sizes were quite small, patients had a history of childhood trauma, and some patients met criteria for PTSD or dissociative disorder (see Table 1).

In another study by Krause-Utz and colleagues [125••], high-frequency heart rate variability (HF-HRV, i.e., a marker of stress regulation) was investigated during an emotion regulation task in female BPD patients with versus without comorbid PTSD and healthy women. Participants were instructed to either attend or downregulate emotional responses to neutral, positive, and negative images. Compared with the other groups, BPD patients with comorbid PTSD showed significantly lower HF-HRV overall. Acute dissociation before the task positively predicted HF-HRV during downregulating versus attending negative pictures in this group. In this study, patients also reported a history of childhood trauma, and some patients met criteria for PTSD. The link between baseline dissociation and HF-HRV during downregulating emotions was limited to the group with comorbid PTSD, which questions the specificity of findings for BPD.

Interestingly, a study by Koenig and colleagues [126••] suggests that dissociation is associated with altered physiological orienting responses to startling sounds in female adolescents (13–19 years) with BPD. Relative habituation of heart rate to acoustic startle probes (sinus tones) was significantly positively correlated with dissociation as well as BPD symptom severity. These findings suggest that dissociative experiences may influence automatic defensive responses early on in the course of BPD [126••].

More research with control groups (clinical groups with dissociative features as well as traumatized individuals who did not develop a disorder) is needed to understand whether certain psychophysiological alterations may be specific to acute dissociation and to BPD. At this point, the number of studies comparing distinct disorders is way too low to draw firm conclusions. The use of specific dissociation measurements (e.g., peritraumatic reactions and acute dissociation as opposed to trait dissociation) may help to improve this understanding.

### Dissociation and Pain Processing

Individuals with BPD show reduced pain perception (higher pain thresholds) compared to healthy control groups, which correlates with emotional distress [127] and dissociation [47, 112]. Reducing acute states of emotional distress and dissociation is one of the most prevalent motives for NSSI in patients with BPD [45, 46].

To investigate if the link between dissociation and reduced pain processing is related to acute presentations of BPD, Chung and colleagues [128••] assessed heat pain thresholds and dissociation ratings in participants with acute BPD, those in remission, and healthy controls. During the abovementioned script-driven imagery paradigm, participants were exposed to either a personalized stressful script or a neutral script (autobiographical narrative). When exposed to the stressful script, patients with acute BPD and those in remission showed enhanced dissociation along with pain hyposensitivity (higher heat pain thresholds). However, a significant association between dissociation and hyposensitivity to pain (analgesia) was only observed in patients with acute BPD, not in those in remission.

In another study by Defrin and colleagues [129], no significant correlation between elevated heat pain thresholds and a measure of trait dissociation (DES) was found in patients with acute BPD. On the other hand, higher trait dissociation (scores on the DES) predicted changes in the default mode network in response to painful heat stimulation in BPD patients with current self-injurious behavior [130]. This suggests that frequent dissociative experiences may be associated with changes in brain regions that underlie processing of pain, e.g., as being less self-relevant or aversive. Future studies may investigate whether alterations in pain processing (e.g., analgesia) are specific to acute dissociative states, as compared to trait dissociation in acute BPD [47, 112, 130].

### Dissociation and Body Ownership

A new avenue of research concerns experiences of body ownership, i.e., the certainty that certain body parts belong to oneself. Alterations in body ownership may contribute to symptoms of depersonalization and somatoform dissociation. Providing first evidence for this idea, a study by Löffler and colleagues [131••] found a significant link between reduced body ownership and higher dissociation when controlling for other BPD core symptoms. In this study, patients with acute BPD experienced lower body ownership than healthy controls, while not differing from those with remitted BPD. Future neurobiological studies may elucidate if reduced body ownership is linked to neurobiological alterations during acute dissociation in BPD and if this is especially the case for those who experienced child sexual abuse [51, 55, 56]. This interesting new research avenue may open up new targets for future research and treatment.

## Conclusion

The aim of this article was to provide an overview of current experimental neurobiological research on dissociation in BPD. Building on our previous review [3], we focused on neuroimaging studies published in 2017 or later. Since experimental research on dissociation in BPD is scarce, we also included psychophysiological and pain processing studies, as well as a study investigating the link between dissociation and body ownership, which were revealed by our literature search in relevant databases.

So far, only a few studies have used experimental paradigms to investigate the impact of dissociation on information processing in BPD [3]. Even fewer studies have directly investigated the impact of acute (i.e., experimentally induced) dissociative states on neural processing. Findings are quite diverse and need to be replicated and should therefore be considered as preliminary. Methodological differences in the measurement of dissociation (e.g., state versus trait dissociation) and analytical techniques complicate the interpretation and comparison of findings. Up to now, it is not entirely understood whether neurobiological changes are relatively stable and linked to general dissociative tendencies, or temporary (i.e., only occurring during acute dissociation).

Moreover, future studies should address the question whether neurobiological changes during dissociation depend on the underlying psychiatric diagnosis, or whether they are transdiagnostic in nature. Functional magnetic resonance imaging studies in BPD found links between dissociative states/traits and altered brain activity in frontal and temporal (limbic-related) regions during symptom provocation tasks and during rest. While altered activity in frontal regions (e.g., dorsomedial and dorsolateral prefrontal cortex, superior frontal regions, anterior cingulate) may be trans-diagnostically linked to dissociation (see previous reviews [4••, 5••]), reduced amygdala activity may be specific to BPD, which needs to be elucidated in future research.

To investigate the disorder-specificity of findings, which is still an open research question, studies need to include distinct psychiatric (control) groups, as the presence of certain comorbid diagnoses may hinder a straightforward interpretation of findings. At the same time, rigor inclusion-/exclusion criteria hamper recruitment, and studies with larger sample sizes as well as meta-analyses of larger data sets are strongly needed to extend and replicate previous findings with sufficient statistical power. Shared etiologies (e.g., trauma history) should be taken into account [132], e.g., by including control groups of participants that experienced trauma without developing psychiatric disorders. Careful screening for childhood trauma, symptom severity, and comorbidities may help to improve the understanding of trauma- and non-trauma-related pathways to dissociation.

With respect to BPD, most studies used aversive stimuli to investigate the impact of dissociation on emotional processing, while dissociation may also interfere with the processing and memory of positive stimuli [66]. Previous studies observed difficulties detecting and memorizing positive social signals and events [57, 62, 63], and discriminating between social inclusion and exclusion [57, 59], which may contribute to difficulties in establishing trust. If dissociation has detrimental effects on processing and memory for both for positive and negative emotions, this may partly explain why acute dissociative symptoms can contribute to poor therapy outcome in patients with BPD [70]. Future neuroimaging studies may investigate dissociation in the context of core symptoms other than emotion dysregulation, such as impulsive and risky decision-making [43] and interpersonal functioning. Shifting the focus to the investigation of interpersonal processes (e.g., trust and face processing) may help to gain more insight into how dissociation may affect interpersonal functioning in BPD.

Increasing knowledge on possible neurobiological markers of dissociation can be an important step in the development and improvement of existing therapeutic, diagnostic, and prognostic approaches [4••, 133]. Individual differences may exist regarding the (psychobiological) markers that predicts treatment outcome [106], and the inclusion of dissociation measures may help to improve this understanding. A combination of multiple measures (e.g., neuroimaging, psychophysiological, self-report), different analytical techniques across different experimental settings, and the inclusion of clinical control groups may be helpful steps in this direction. Thereby, future studies may also investigate if clinical interventions aimed at reducing dissociation are associated with a normalization of functional brain alterations.
